# A comparison of oral health, nutrition, and swallowing function in older adults with and without sarcopenia: A cross‐sectional study

**DOI:** 10.1002/ncp.11283

**Published:** 2025-03-04

**Authors:** Melike Yücel, Nezehat Özgül Ünlüer, Yasemin Ateş Sari

**Affiliations:** ^1^ Zirve Special Education and Rehabilitation Center Diyarbakır Turkey; ^2^ Gülhane Faculty of Physiotherapy and Rehabilitation University of Health Sciences Ankara Turkey; ^3^ Department of Physiotherapy and Rehabilitation, Faculty of Health Sciences Ankara Yıldırım Beyazıt University Ankara Turkey

**Keywords:** nutrition, old age, oral health, sarcopenia, swallowing

## Abstract

**Background:**

Sarcopenia, the age‐related loss of muscle mass and strength, may impact the muscles involved in oral functions and swallowing, leading to challenges that may impact quality of life in older adults. The aim of the study was to compare oral health, swallowing function, and malnutrition of older adults with and without sarcopenia.

**Materials and Methods:**

The cross‐sectional study included volunteers ≥65 years of age. Sarcopenia status was evaluated by anthropometric (calf circumference and midupper arm circumference) and muscle strength (walking speed and handgrip strength) measurements. Oral health was assessed with the Oral Health Impact Profile (OHIP‐14), swallowing function was assessed by the Eating Assessment Tool‐10 (EAT‐10), and nutrition status was determined using the Mini Nutritional Assessment.

**Results:**

This study included 65 older adults. The mean age was 80 years, 54% were female, and 43% were diagnosed with sarcopenia. Individuals with sarcopenia had a higher OHIP‐14 score (which indicates poor quality of life related to oral and dental health, 16 ± 8 vs 11 ± 7; *P* = 0.008), were more likely to have a EAT‐10 score ≥ 3 (indicating presence of dysphagia, 79% vs 41%; *P* = 0.002), and were more likely to be at risk for malnutrition (79% vs 54%; *P* = 0.03) compared with individuals without sarcopenia.

**Conclusions:**

Older adults with sarcopenia may be at risk for poor quality of life related to oral health, malnutrition and dysphagia. Further studies with long‐term follow‐up are needed to determine the long‐term effects of sarcopenia on oral health, swallowing function, and malnutrition in older adults.

## INTRODUCTION

The population of older adults in Turkey is increasing in accordance with the global trend; the worldwide population of older adults is projected to reach around 2.1 billion by 2050.^1^ With advancing age, health problems among older adults become more prevalent.^2^, ^3^ Older individuals may experience falls, pressure ulcers, urinary incontinence, dementia, depression, polypharmacy, malnutrition, and frailty.^4^ Sarcopenia, which is characterized by widespread and progressive loss of skeletal muscle and decreased muscle strength and function, is common in older adults and negatively affects the functional independence. Although the cause of sarcopenia is unknown, it develops because of many factors such as aging, malnutrition, immobility/sedentary life, chronic diseases, and drug use.^5^

In this population, oral care and swallowing function may deteriorate because of age‐related changes in anatomical structures and physiological function.^6^ Poor oral hygiene, cavities, periodontal disease, and ill‐fitting dentures are common in this population. The distribution and number of teeth affect chewing comfort and the availability of dental prostheses. Tooth loss can lead to a limited selection of foods and changes in the types of food chosen based on their consistency. As a result, it can reduce appetite, resulting in loss of pleasure in eating, which is considered a risk factor for malnutrition.^7^ Oral health problems can have negative effects on poor diet quality and micronutrient and macronutrient intake.^8^ Additionally, poor nutrition quality has been reported to be associated with poor oral health later in life.^7^, ^8^ This supports a bidirectional relationship between poor oral health and nutrition.

Sarcopenia is a whole‐body process that affects not only the lower extremities but also the muscles dedicated to breathing, chewing, and swallowing.^9^ Swallowing, in particular, is a complex mechanism that involves several head and neck muscles working simultaneously to coordinate the entire process. Various age‐related changes, such as decreased tissue elasticity, changes in head and neck anatomy, decreased oral and pharyngeal sensitivity, and deterioration of dental health, may contribute to mild swallowing impairment. This may increase the risk of dysphagia and aspiration in older adults, especially during acute illnesses and other stressors.^10^ Swallowing dysfunction can occur anywhere in the process from the moment food enters the mouth until it passes into the stomach. It can be seen in all age groups because of multiple congenital, mechanical, structural, neurological, tumor and psychological conditions. Specifically, older adults may experience decreased chewing muscle capacity, reduced tongue strength, delayed formation of the swallowing reflex, reduced salivation, and dry mouth because of drug use.^11^

Sarcopenia may exacerbate these issues with oral care and swallowing function. Moreover, impaired oral health can have a significant impact on nutrition status and can lead to malnutrition.^12^ There are studies in the literature examining oral health and swallowing function in older adults with sarcopenia.^13^, ^14^, ^15^ The distinction of our study is that it encompasses both older adults with and without sarcopenia, and it examines oral health, nutrition, and dysphagia collectively. The hypothesis of this study is that older adults with sarcopenia will exhibit greater impairment in oral health, nutrition status, and swallowing function compared with those without sarcopenia.

## MATERIALS AND METHODS

### Study design and participants

This cross‐sectional study was conducted between May 2021 and May 2022 in the participants' homes. In this study, community‐dwelling older adults were reached via the snowball sampling method. Initially, primary participants representing the target population of the study were identified from the researchers' network. Subsequently, the primary participants were asked to extend an invitation to other potentially eligible individuals. The study was approved by the University Ethics Committee (2021‐05). All participants were informed of the purpose of the study and provided written informed consent before participation. The study was conducted in accordance with the tenets set forth in the Declaration of Helsinki.

Inclusion criteria for the older adults in this study were: (a) being ≥65 years of age (b) having a cognitive level above 24 points according to Mini‐Mental State Examination^16^ (c) being physical independence in activities of daily living. Individuals with any history of neurological disease, degenerative neuromuscular disease, and/or previous surgical and/or radiation therapy to the head and neck were excluded from participation in the study.

### Measurements

Participants' age, body mass index, sex, education level, occupation, dominant extremity, dominant chewing side, number of falls in the previous year, medication use, and assistive device use were recorded. The dominant extremity was identified through a combination of patient self‐report and observational analysis of their writing performance. The dominant chewing side was identified through a series of steps. Initially, the participants were instructed to chew gum and their dominant chewing side was observed. Subsequently, the frequency of falls experienced by the participants over the previous six months was documented. Additionally, the use of assistive devices that could potentially impact balance or the occurrence of falls was recorded.

The assessment tasks (anthropometric measurement, walking speed, handgrip strength, oral health, nutrition, and swallowing function) were initially presented to the participants by the assessor, who provided a detailed explanation and demonstration of each step. The same physiotherapist (MY) was responsible for the measurements. The assessments were conducted concurrently in a tranquil, well‐lit room. Participants were permitted a 2‐min intermission between measurements to preclude the potential effects of fatigue.

The MMSE, which has been validated in Turkish and demonstrates reliability, was employed to assess cognitive status. The MMSE is a battery of tests that assesses various cognitive functions, including memory, orientation, calculation, recall, attention, language, perceptual ability, and motor function. The maximum score is 30. A score of ≤23 is indicative of cognitive impairment.^16^

Sarcopenia was assessed according to an algorithm developed by the European Working Group on Sarcopenia in Older People (EWGSOP).^3^ Walking speed was used to define muscle function, handgrip strength was used to define muscle power, and calf circumference and upper arm circumference were used to define muscle mass. Both walking speed and handgrip strength were measured in the current study. Accordingly, this algorithm posits that participants exhibiting a combination of reduced walking speed and low muscle mass, or normal walking speed and weakness in both grip strength and muscle mass are to be defined as individuals afflicted with sarcopenia.

To assessing walking speed, the individual was requested to traverse a distance of six meters at a pace consistent with their typical daily gait. The walking speed was subsequently calculated in meters per second by noting the time taken to complete the aforementioned distance. A walking speed of <0.8 meters per second was deemed to indicate poor physical performance.

Handgrip strength was measured using a Jamar hand dynamometer (Baseline Evaluation System). In a seated position, with the tested arm in shoulder adduction, the elbow in 90° flexion, the forearm in midrotation and the wrist in neutral, the participant was instructed to exert maximal force upon the dynamometer and then to allow the hand to relax completely.^17^ The measurements for both the right and left hands were taken on three occasions, and the mean of these three values was recorded. A value of <20 kg for women and <30 kg for men was deemed indicative of poor muscle strength.^3^

Measurements of calf circumference and upper arm circumference were used to determine muscle mass. Calf circumference was measured at the widest point of the calf, with the ankle and knee positioned at a 90° angle while the participant was seated. A calf circumference of <31 cm was interpreted as poor muscle mass.^18^ Upper arm circumference was measured with a tape measure from the midpoint between the shoulder and elbow protrusion, with the arms at a 90° angle and palms facing each other.^19^ Values <22 cm for women and <23 cm for men were considered to be indicative of poor muscle mass.^20^

The Charlson Comorbidity Index, which is a commonly employed tool for the assessment of comorbid conditions and has been validated for the prediction of mortality, was utilized. Information on participants’ comorbid conditions was obtained from their medical records. The total score is calculated based on these scores:

- 1 point: the presence of congestive heart failure, myocardial infarction, cerebrovascular disease, peripheral vascular diseases, dementia, chronic pulmonary disease, ulcers, connective tissue, mild liver disease and diabetes.;
- 2 points: the presence of moderate/severe renal disease, hemiplegia, diabetes causing end organ damage, leukemia, tumor presence in the presence of malignant lymphoma;
- 3 points: the presence of moderate/severe liver disease; and
- 6 points: the presence of AIDS and metastatic solid tumor; mortality risk increases as the comorbidity score increases.^21^

The Oral Health Impact Profile‐14 (OHIP‐14) was developed for the purpose of evaluating deficiencies and discomforts associated with oral and dental health. The scale assesses a range of factors that contribute to an individual's quality of life, including functional limitations, physical and psychological discomfort, and social inadequacy. The questions in the tool were asked to the participants by the physiotherapist (M.Y.) and the answers to each were recorded. A high score on the scale is indicative of poor oral and dental health‐related quality of life.^22^

The Eating Assessment Tool‐10 (EAT‐10) was used to evaluate swallowing function. The questions in the tool were asked to the participants by the physiotherapist (MY) and the answers to each were recorded. In the 10‐item questionnaire, each item is scored by individuals on a scale from “0” (no problem) to “4” (serious problem). and the total score is recorded. A total score of 3 and above indicates the presence of dysphagia.^23^

Nutrition status assessment was performed with the Mini Nutritional Assessment Test (MNA). The MNA consists of four parts including anthropometric assessment, general assessment, brief nutrition assessment, and subjective assessment.^24^ Nutrition status of the individuals was evaluated as no malnutrition (≥24), at nutrition risk (between 17 and 23.5), and malnourished (<17).^24^ The same physiotherapist (M.Y.) was responsible for the evaluation.

### Statistical analysis

The Statistical Package for Social Sciences (SPSS), version 22, was used for statistical evaluation of the data. The normality of continuous variables was evaluated using the Shapiro‐Wilks test. For continuous variables, median (minimum to maximum) and mean ± standard deviation values are given, whereas number and percentage values are given for categorical variables. For comparisons of sarcopenia and without sarcopenia groups, the independent two‐sample *t* test was used for normally distributed data (walking speed, OHIP‐14) and the Mann Whitney *U* Test was used for nonnormally distributed data (other data). Categorical data (sex, education level, working status, dominant extremity and chewing, use of assistive device, nutrition status, presence of dysphagia) were analyzed using the chi‐square test or the Fisher exact test. Statistical significance level was accepted as *P* < 0.05.

Post hoc power analysis was performed using the G* Power program (version 3.0.10 Universität Düsseldorf, Düsseldorf, Germany). The current study observed a significant difference in EAT‐10 scores between the group with sarcopenia (*n* = 28) and those without sarcopenia (*n* = 37), with an effect size of 0.89.

## RESULTS

The study was completed with 65 older adults, 28 with sarcopenia (15 women, 13 men; 82 ± 7 years) and 37 without (20 women, 17 men; 80 ± 6 years). As seen in Table 1, the group without sarcopenia exhibited a higher body mass index compared with individuals with sarcopenia (29.83 ± 4.05 kg/m² vs 23.56 ± 3.25 kg/m², *P* < 0.05) (Table 1). The age, sex, educational level, working status, and Charlson Comorbidity Index of the participants included in the study were found to be similar (*P* > 0.05).

**Table 1:** Comparison of demographic information of older adults.

Variables	Group with sarcopenia (*n* = 28)	Group without sarcopenia (*n* = 37)	*P*
Age, mean ± SD, year	82 ± 7	80 ± 6	0.261
BMI, mean ± SD, kg/m^2^	23.56 ± 3.25	29.83 ± 4.05	<0.001*
Sex, *n* (%)			
Female	15 (53.6)	20 (54.1)	0.969
Male	13 (46.4)	17 (45.9)	
Education level, *n* (%)			
Primary school	21 (75)	31 (83.8)	0.381
Secondary School	2 (7.1)	4 (10.8)	
High school	4 (14.3)	2 (5.4)	
University	1 (3.6)	0	
Working status, *n* (%)			
Working	0	0	0.969
Retired	13 (46.4)	17 (45.9)	
Not working	15 (53.6)	20 (54.1)	
Charlson comorbidity index	1 (0.3)	2 (0.5)	0.110

No statistically significant differences were observed between the groups with regard to the dominant limb, the dominant chewing side, the use of assistive devices, the number of medications used, the frequency of falls, and grip strength (*P* > 0.05). The group without sarcopenia exhibited superior cognitive status (*P* < 0.05; Table 2).

**Table 2:** Comparison of descriptive characteristics of older adults.

Variables	Group with sarcopenia (*n* = 28)	Group without sarcopenia (*n* = 37)	*P*
Dominant extremity, *n* (%)			
Right	27 (96.4)	35 (94.6)	1^a^
Left	1 (3.6)	2 (5.4)	
Dominant chewing, *n* (%)			
Right	28 (100)	36 (97.3)	1^a^
Left	0	1 (2.7)	
Use of assistive devices, *n* (%)			
Glasses	8 (28.6)	16 (43.2)	0.429
Hearing aid	1 (3.6)	2 (5.4)	
Cane	12 (42.9)	9 (24.3)	
None	7 (25)	10 (27)	
Number of medicines used, mean (SD)	3 (1.7)	4 (0.13)	0.208
Frequency of falls, mean (SD)	0 (0.4)	0 (0.3)	0.182
Mini‐mental state examination, mean (SD)	25 (24.30)	27 (24.30)	0.003
Gripping force, median (minimum to maximum), kg			
Right	18.60 (4.00–42.60)	18.66 (11.50–43.33)	0.199
Left	17.30 (4.00–43.00)	19.33 (7.83–40.50)	0.230
Arm circumference measurement, median (minimum to maximum), cm			
Right	21.50 (18.00–22.50)	31.20 (25.30–41.00)	<0.001
Left	21.50 (19.00–22.60)	30.00 (25.50–38.50)	<0.001
Calf circumference measurement, median (minimum to maximum), cm			
Right	29.45 (25.60–30.80)	36.50 (32.00–42.00)	<0.001
Left	29.50 (25.20–30.70)	35.60 (31.30–41.00)	<0.001
Walking speed, mean ± SD, m/s	0.52 ± 0.21	0.65 ± 0.21	0.015

^a^ Fisher Exact Test, *P* < 0.05.

Significant differences were observed in oral health, nutrition status, and swallowing function between the groups (*P* < 0.05). The subparameters of the OHIP‐14 questionnaire were analyzed, and it was found that the group with sarcopenia exhibited higher levels of physical pain, psychosocial inadequacy, and social inadequacy. With regard to the nutrition status of the group with sarcopenia, 10.7% were identified as malnourished, 67.9% were classified as being at risk, and 21.4% exhibited normal nutrition status. In the cohort without sarcopenia, no individuals were malnourished, 54.1% were at risk, and 45.9% had normal nutrition status. In addition, the median EAT‐10 score was 5 in the group with sarcopenia and 2 in the in the group without sarcopenia. Regarding swallowing functions in relation with the EAT‐10 score, 78.6% in the group with sarcopenia and 40.5% in the group without sarcopenia exhibited a score of ≥ 3 (Table 3).

**Table 3:** Comparison of oral health, nutrition status and swallowing function of patients with and without sarcopenia.

Variables	Group with sarcopenia (*n* = 28)	Group without sarcopenia (*n* = 37)	*P*
OHIP‐14, score, median (minimum to maximum)			
Functional constraint	4 (0–6)	2 (0–8)	0.071
Physical pain	4 (0–6)	3 (0–8)	0.018
Psychological discomfort	4 (0–7)	4 (0–8)	0.315
Physical inadequacy	3 (0–6)	3 (0–8)	0.123
Psychosocial inadequacy	1 (0–4)	0 (0–5)	0.002
Social inadequacy	1 (0–6)	0 (0–4)	<0.001
Handicap	0 (0–1)	0 (0–0)	0.101
Total	16 ± 8	11 ± 7	0.008
MNA test			
MNA score, median (minimum to maximum)	21.00 (12.50–27.00)	23.50 (18.00–27.50)	0.004
Normal, n (%)	6 (21.4)	17 (45.9)	0.028
At risk, n (%)	19 (67.9)	20 (54.1)	
Malnourished, n (%)	3 (10.7)	0	
EAT‐10			
Score, median (minimum to maximum)	5 (0–16)	2 (0–14)	<0.001
<3, n (%)	6 (21.4)	22 (59.5)	0.002
≥3, n (%)	22 (78.6)	15 (40.5)	

Abbreviations: EAT‐10, Eating Assessment Tool; MNA, Mini Nutritional Assessment Test; OHIP‐14, Oral Health Impact Profile.

## DISCUSSION

In this study, we compared the oral health, nutrition status, and swallowing function of older adults with and without sarcopenia, and found that older adults with sarcopenia had poorer oral health and nutrition status and higher swallowing dysfunction than those without sarcopenia, confirming our hypothesis.

The risk of sarcopenia increases with advancing age.^25^ As the body ages, a series of irreversible physiological, structural, and functional changes occur at the molecular, cellular, tissue, organ, and system levels.^3^ It is notable that changes in oral health, nutrition and swallowing status are particularly prevalent in older adults with sarcopenia or at risk of sarcopenia.^26^ The results of our study demonstrated that the total OHIP‐14 score and the scores for the subparameters of physical pain, psychosocial inadequacy and social inadequacy differed between the groups. The elevated scores observed in individuals with sarcopenia suggest that older adults with sarcopenia are more prone to experiencing poorer oral health outcomes. This topic has been extensively investigated in the scientific literature.^26^, ^27^ Takahashi et al.^27^ observed a prevalence of sarcopenia among outpatient older adults of 30.8% and reported a positive association between sarcopenia and poorer oral health. Furthermore, Shiraishi et al.^26^ indicated that an inferior oral health status in poststroke patients was linked to the development of sarcopenia. In their review of the relationship between oral health and sarcopenia, Hatta and Ikebe^14^ report that poor oral health has a negative impact on dietary intake, leading to lower levels of intake of multiple nutrients, including protein and calcium. It is hypothesized that a deficiency of these nutrients may be a significant contributing factor to the reduction in muscle mass, muscle strength and physical performance. This suggests that poor oral health may contribute to the development of sarcopenia by negatively impacting an individual's nutrition habits.^28^, ^29^ In conclusion, given the disparate methodologies employed in the existing literature on oral health, our findings align with those of previous studies. Poor oral health may lead to malnutrition and loss of grip strength in older adults with sarcopenia. Therefore, it is one of the parameters that should not be ignored in individuals with sarcopenia.

Malnutrition represents a significant clinical concern among older adults. Aging, illness, multiple drug use, oral dysfunction, difficulty in shopping, difficulty in preparing meals, and economic problems are among the environmental factors that contribute to malnutrition in older adults.^30^, ^31^, ^32^ The findings of our study indicate that 21.4% of individuals in the group with sarcopenia had normal nutrition intake, 67.9% were at risk of malnutrition, and 10.7% were malnourished. In the group without sarcopenia, no individuals were malnourished. However, 48.9% of the group was at risk. The coexistence of malnutrition and sarcopenia has been previously documented in the scientific literature. These studies have included both inpatients and outpatients, employing diverse methodologies and assessment tools for the evaluation of sarcopenia and malnutrition in older adults.^33^ In a study conducted by Ülger et al.,^33^ it was found that 28% of outpatients presenting at the geriatric outpatient clinic exhibited poor nutrition status. Among inpatients, the prevalence of individuals at risk of malnutrition was 69%, whereas the prevalence of those who were malnourished was 12%. In a study conducted by Liguori et al.,^34^ the prevalence of malnutrition and sarcopenia in older adults was evaluated. The findings indicated that the overall prevalence of sarcopenia was 13.1%, with an increase from 6.1% to 31.4% observed as the MNA score decreased. It has been reported that older adults with sarcopenia exhibit poor nutrition status, and that the MNA score is linearly correlated with both muscle mass and muscle strength, despite the presence of several confounding variables, including comorbidity and disability.^34^ Our findings corroborate the existing literature by indicating that the risk of malnutrition is also elevated in older adults at risk of sarcopenia. In this context, they underscore the importance of comprehensive malnutrition assessment in older adults from an early stage.

Sarcopenia can result in physical impairment, such as dysphagia. Furthermore, malnutrition, which may be a consequence of dysphagia, may also lead to sarcopenia. It is therefore posited in the literature that there is a cyclical relationship between sarcopenia, nutrition, swallowing and oral health conditions, although the precise underlying mechanism remains unknown.^35^ In a systematic review to determine the relationship between sarcopenia and dysphagia, Zhao et al.^36^ reported a positive correlation between the two conditions, indicating that individuals with sarcopenia tend to have poorer swallowing function. Nevertheless, it remains uncertain whether dysphagia is a consequence of sarcopenia or whether sarcopenia is a consequence of dysphagia. In their study of 236 older adults, Cha et al.^37^ observed that individuals with sarcopenia exhibited a significantly elevated prevalence of dysphagia. The prevalence of dysphagia was 78.5% in the sarcopenic group and 42.6% in the nonsarcopenic group, as observed in our study. The observation that individuals with sarcopenia exhibited greater swallowing impairment than those without sarcopenia suggests a mutual influence between sarcopenia and swallowing. These findings are in accordance with the existing literature on the participant.^38^, ^39^ Nutrition disorders and changes in swallowing function may result in adverse effects on vital functions in older adults with sarcopenia. It is therefore crucial to identify this condition in primary care.

The present study revealed that individuals with sarcopenia exhibited lower body weight and body mass index (BMI) compared with those without sarcopenia. A number of studies have demonstrated that individuals with sarcopenia tend to exhibit a lower body mass index (BMI) compared with those without sarcopenia.^40^, ^41^ Additionally, research has indicated that individuals with sarcopenia tend to exhibit a higher body mass index (BMI) compared with those without this condition.^42^, ^43^ This case also demonstrates the necessity of considering sarcopenic obesity in older adults.

Our study also revealed a difference in cognitive performance between the group with and without sarcopenia. In their study of women >75 years of age, Abellan van Kan et al.^44^ found no correlation between sarcopenia and cognitive impairment. In their systematic review and meta‐analysis, Peng et al.^45^ identified an association between sarcopenia and both mild cognitive impairment and dementia. A comparative analysis of older adults with and without sarcopenia revealed that the risk of mild cognitive impairment and dementia was approximately doubled in the presence of sarcopenia.^45^ Furthermore, this emphasizes the significance of prompt diagnosis of sarcopenia in order to avert cognitive decline.

It must be acknowledged that our study was not without limitations. One such limitation was that it was not possible to group older adults with sarcopenia according to the severity of the condition; this would have included presarcopenia, sarcopenia, and severe sarcopenia. In addition, the swallowing function was assessed using the subjective EAT‐10. The inclusion of individuals with varying degrees of sarcopenia in studies utilizing the videofluoroscopic method, which represents the gold standard for swallowing assessment, will facilitate the generation of more objective results.

## CONCLUSION

The findings revealed that older adults with sarcopenia exhibited inferior oral health and nutrition status, along with a greater prevalence of swallowing dysfunction, in comparison with those without sarcopenia. In light of the aforementioned changes associated with sarcopenia, it is imperative to assess an individual's oral health, nutrition status, and swallowing function in order to gain insight into their underlying risk factors and to facilitate the early detection of potential complications. In addition, considering the increase observed in the elderly population in recent years, early evaluation of these parameters and appropriate interventions can contribute to the support of healthy aging and thus to the prevention of age‐related conditions such as sarcopenia, oral health problems, feeding, and swallowing difficulties.

## AUTHOR CONTRIBUTIONS

Melike Yücel and Nezehat Özgül Ünlüer contributed to the conception and design of the research. Melike Yücel and Yasemin Ateş Sari contributed to the acquisition and analysis of the data. Nezehat Özgül Ünlüer and Yasemin Ateş Sari contributed to the interpretation of the data. Melike Yücel and Yasemin Ateş Sari drafted the manuscript. All authors critically revised the manuscript, agree to be fully accountable for ensuring the integrity and accuracy of the work, and read and approved the final manuscript.

## CONFLICT OF INTEREST STATEMENT

None declared.
